# Delayed Splenic Rupture Resulting in Massive Intraperitoneal Hemorrhage Post Ambulatory-Related Injury

**DOI:** 10.7759/cureus.2160

**Published:** 2018-02-05

**Authors:** Chikamuche T Anyanwu, Shane D Reynal

**Affiliations:** 1 Surgery, Texas A&M College of Medicine; 2 College of Medicine, Texas A&M College of Medicine

**Keywords:** delayed splenic rupture, blunt abdominal trauma, computed tomography, splenic rupture, minor trauma, non-operative management

## Abstract

Delayed splenic rupture, once thought to be unusual, but now growing in incidence, is an issue that could potentiate severe morbidity and mortality to patients, regardless of the severity of the trauma. This case report presents one instance of delayed splenic rupture following minor trauma and discusses the need for further investigation in the management of this condition.

A middle-aged, hypotensive male presented to the emergency department (ED) with signs of an acute abdomen following a syncopal episode, with successful resuscitation upon arrival. Computed tomography (CT) revealed fluid in the gastrohepatic ligament, as well as the right pericolic gutter, along with findings suggestive of a perforated peptic ulcer. General surgery was consulted, and an exploratory laparotomy was performed. However, during exploration, no such perforation was found. A large amount of blood was visualized in the left upper quadrant. The spleen was mobilized, and inspection revealed a posterior rupture, resulting in a splenectomy and subsequent hemostasis by the surgical team. The post-operative period was uneventful, with the patient revealing a previous fall onto a concrete floor two weeks prior to presentation to the ED. A pathological examination of the spleen revealed capsular tear with focal congestion and hemorrhage.

It is the goal of this report to highlight the morbidity and mortality that occur after delayed splenic rupture, even with minor traumatic events. Current guidelines suggest nonoperative management of splenic injury in stable patients with low-grade splenic injuries after blunt abdominal trauma. However, with the varied presentation and difficulty in the assessment of delayed splenic rupture, patients may be exposed to undue risks with current recommendations on the management of splenic injuries. Further research is needed to find the best practice in managing, and possibly preventing, delayed splenic rupture in patients presenting with an acute abdomen or abdominal trauma.

## Introduction

The liver and spleen are the solid organs most commonly injured from blunt abdominal trauma. Although nonoperative management of hepatic injuries is the treatment of choice in the hemodynamically stable patient, the management of splenic injury following blunt abdominal trauma is highly subjective, with treatment usually based on the grading scale published by the American Association for the Surgery of Trauma (AAST). This score ranges from subcapsular hematoma (Grade 1) to a shattered spleen (Grade V) [1]. Splenic injury in the traumatic setting may present as either subcapsular or intraparenchymal hematomas or as vascular lacerations. Complete splenic obliteration may lead to significant hemodynamic instability and subsequent shock. Pseudocysts, pseudoaneurysms, arteriovenous fistulas, and delayed splenic rupture are possible complications of splenic injury [2].

Splenic rupture presents in either of two ways: acutely or in a delayed presentation. Delayed splenic rupture was first described in the early 1900s by Baudet as bleeding occurring more than 48 hours post blunt abdominal trauma [3].Though largely unknown, it is postulated that delayed splenic rupture may arise from splenic parenchymal pseudoaneurysm formation, in which clot dissolution contributes to the degradation of the aneurysmal wall, leading to delayed hemorrhaging [3]. Initially, most splenic ruptures were treated with exploratory laparotomies/surgical intervention with the notion that delayed splenic rupture was an unusual complication of blunt abdominal trauma and that the delayed presentation was, in fact, a delayed recognition of an acute splenic rupture [4]. However, in the 1970s, according to Friedwald, a more conservative approach was adopted for the observation of patients. This has been endorsed by the Eastern Association for the Surgery of Trauma (EAST) in the practice management guidelines for nonoperative management of blunt injury to the liver and Spleen [1]. However, one of the possibilities with the nonoperative management of splenic injury is the presentation of delayed splenic rupture, leading to increased morbidity and mortality. We present a case of delayed splenic rupture, not in an acute setting, where the patient could have experienced severe morbidity and mortality due to significant intraperitoneal hemorrhage.

## Case presentation

**Figure 1:**
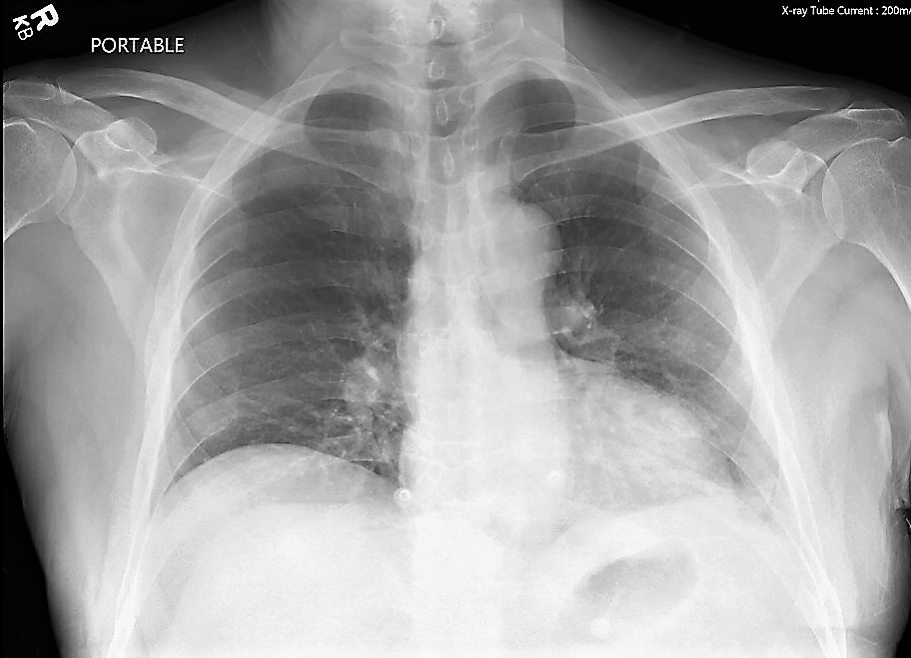
Anterior-posterior chest radiograph showing no acute cardiopulmonary process

A 62-year-old Hispanic male presented to the emergency department (ED) via emergency medical services (EMS) for a syncopal episode. He was found to be hypotensive and tachycardic and was successfully resuscitated en route to the ED. He reported a sudden onset of diffuse abdominal pain, nausea, vomiting, diaphoresis, and urinary hesitancy. Evaluation in the ED revealed bilateral nystagmus, decreased diffuse breath sounds, abdominal rigidity with rebound tenderness, and left upper quadrant guarding. No chest pain, fever, chills, numbness, or tingling was reported. Vital signs included a temperature of 97.8°F, pulse of 104, respiratory rate of 18, oxygen saturation of 92%, and blood pressure of 105/69. He underwent a chest x-ray (Figure 1), computed tomography (CT) brain, and computed tomography angiography (CTA) thorax and abdomen with contrast. Fluid was noted in the gastrohepatic ligament with questionable extraluminal air. Additionally, fluid was evident around the liver and down the right pericolic gutter. The patient denied any history of peptic ulcer disease. He was a nonsmoker, with no history of alcohol consumption.

**Figure 2:**
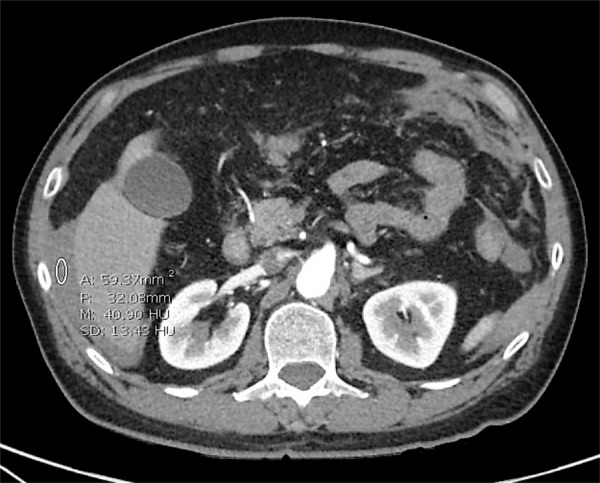
Transverse section on CT abdomen showing free fluid around the liver and subcapsular hepatic cyst CT, computed tomography

**Figure 3:**
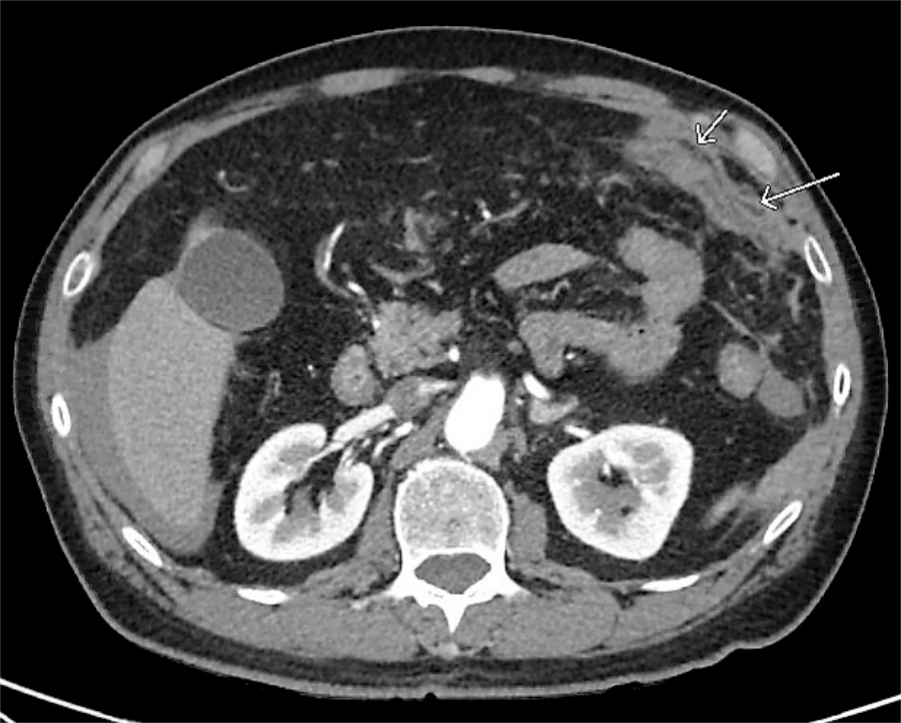
Transverse section on CT abdomen showing soft tissue attenuation with surrounding fat stranding consistent with hematoma CT, computed tomography

The patients’ past medical history was significant for hyperlipidemia and hypertension. Surgical history was significant for inguinal hernia repair. Medications included the chronic use of aspirin 325 mg for subjective arthritic pain. Laboratory findings included leukocytosis (15.8 thou/µL) and hemoglobin (14.4). CT findings indicated a preoperative diagnosis of a perforated peptic ulcer with the Hounsfield units of the fluid suggestive of blood (Figures 2-3). General surgery was consulted for emergency exploration.

Exploratory laparotomy revealed no evidence of a perforated ulcer, peptic or otherwise. There was evidence of hepatic hemangiomas, as identified on CT, but no evidence of their rupture, liver injury, or lacerations. There was an excessive amount of blood around the left upper quadrant. The spleen was mobilized and inspected, which revealed a posterior rupture. The spleen was subsequently removed and hemostasis confirmed. No other abnormalities were noted during the surgery. Estimated blood loss was 600 mL (400 mL of old blood and 200 mL of new blood).

Further patient questioning during the postoperative period revealed a previous fall onto concrete while at work two weeks prior to presentation. No medical attention was sought at that time, as the patient believed the traumatic event was too trivial to warrant medical attention. Post-operative recovery was uneventful, and the patient was discharged three days post-op. Pathological examination of the spleen revealed a weight of 168 grams with attached fat. The spleen measured 12.4 x 7.8 x 1.3 cm. Two visible torn areas, measuring 2.2 cm and 4.2 cm, were noted. There were also two hemorrhagic cystic areas, measuring 1.5 cm and 4.5 cm, on the serosal surface. A microscopic analysis of the extricated spleen revealed areas of congestion and hemorrhage.

## Discussion

According to the American College of Surgeons (ACS), mortality from splenic injuries accounts for approximately 10% of all trauma-related deaths [5]. In 1998, Cocoanour stated that the incidence of delayed splenic injury was approximately 1% [6]. However, in 2017, Furlan et al. reported that the incidence of delayed splenic injury has risen to 3%-15% with an inconsistent association with the severity of splenic trauma [7]. Compared to the 1% mortality associated with acute splenic injury, the mortality from delayed diagnosis of splenic injury following blunt abdominal trauma ranges from 5%-15% [3]. The trend in the increased presentation of delayed splenic injury is a risk of patient morbidity and mortality and can be attributed to the difficulty in identifying delayed splenic rupture. Difficulties with diagnosing delayed splenic injury may include atypical presentations that may mimic other pathologies, the lack of symptomatology, or equivocal imaging. Due to the variability in presentation and the difficulty with the initial assessment of delayed splenic injury, it is the opinion of the authors that delayed splenic rupture be considered in the differential diagnosis of an acute surgical abdomen regardless of the time or mechanism of the inciting event.

Current Advanced Trauma Life Support (ATLS) guidelines from the ACS regarding the management of splenic injury following trauma includes observation, embolization, or surgical intervention based on the hemodynamic status of the patient. Management is less subjective in the hemodynamically unstable patient, in which surgical intervention is uniform. Controversy or subjective management may compromise best practice in the otherwise hemodynamically stable patient with seemingly minor injuries who is at risk of delayed splenic rupture. Patients with splenic injuries without evidence of acute extravasation are usually managed nonoperatively. Despite the higher rates of success with the conservative management of patients with low-grade splenic injuries, given the increase in the use of this approach, there should be a concern that current practice may lead to increased mortality in patients presenting with complications from nonoperative management.

One area of controversy in the management of splenic insults is the use of serial CT scans as an adjunct to the nonoperative management of splenic injury. Weinberg et al. reported high splenic salvage in patients with pseudoaneurysms who underwent conservative management with serial CT surveillance. They recommended the use of serial CT scans in all patients undergoing the nonoperative management of splenic injury regardless of grade of injury [8]. However, EAST guidelines have found no evidence to support routine imaging (CT or ultrasound) in hemodynamically, clinically improving patients [1].

Notwithstanding the favorable outcomes following continued surveillance in patients with the nonoperative management of splenic injuries, an expert survey showed that 88% of clinicians do not recommend post-discharge imaging for patients managed conservatively [9]. We recognize that the feasibility of serial CT surveillance may not be cost-effective and may be hindered by patient adherence, co-morbid conditions, and exposure to radiation. Thus, alternative considerations should be given to ensure prompt diagnosis and effective, both acutely and long-term, management of delayed splenic rupture. It should be of note that Lui et al. compared the effectiveness of operative and nonoperative management of splenic ruptures and concluded that quality of life post splenic rupture was similar, though patients who had undergone nonoperative management did experience longer hospital stays [10]. Hence, there may be some benefit of early splenectomy even in low-grade splenic injuries.

## Conclusions

The purpose of this report is to emphasize the importance of accurate history taking in the evaluation of a patient with an acute abdomen as well as to highlight delayed splenic rupture following relatively minor trauma. This report also highlights a possible complication or subsequent morbidity of nonsurgical intervention for even mild degrees of splenic rupture. Given the paucity of data on delayed splenic rupture and the availability of evidence limited to mostly case reports, further study is needed to modify existing recommendations regarding the initial and subsequent management of splenic injuries that may be fatal in the long run.
